# Psychological Distress and Anxiety Levels Among Health Care Workers at the Height of the COVID-19 Pandemic in the United Arab Emirates

**DOI:** 10.3389/ijph.2021.1604369

**Published:** 2021-11-11

**Authors:** Basema Saddik, Iffat Elbarazi, Mohamad-Hani Temsah, Fatemeh Saheb Sharif-Askari, Waad Kheder, Amal Hussein, Hellme Najim, Riyad Bendardaf, Qutayba Hamid, Rabih Halwani

**Affiliations:** ^1^ Department of Family and Community Medicine, College of Medicine, University of Sharjah, Sharjah, United Arab Emirates; ^2^ Sharjah Institute of Medical Research, University of Sharjah, Sharjah, United Arab Emirates; ^3^ Institute of Public Health, College of Medicine and Health Sciences, United Arab Emirates University, Al-Ain, United Arab Emirates; ^4^ Department of Pediatrics, College of Medicine, King Saud University, Riyadh, Saudi Arabia; ^5^ Prince Abdullah Ben Khaled Celiac Disease Chair, College of Medicine, King Saud University, Riyadh, Saudi Arabia; ^6^ College of Dental Medicine, University of Sharjah, Sharjah, United Arab Emirates; ^7^ University Hospital Sharjah (UHS), Sharjah, United Arab Emirates; ^8^ Department of Clinical Sciences, College of Medicine, University of Sharjah, Sharjah, United Arab Emirates

**Keywords:** mental health, healthcare workers, COVID-19, psychological distress, anxiety disorders

## Abstract

**Objectives:** Providing medical care during a global pandemic exposes healthcare workers (HCW) to a high level of risk, causing anxiety and stress. This study aimed to assess the prevalence of anxiety and psychological distress among HCWs during COVID-19.

**Methods:** We invited HCWs from 3 hospitals across the United Arab Emirates (UAE) to participate in an anonymous online survey between April 19–May 3, 2020. The GAD-7 and K10 measures were used to assess anxiety and psychological distress. Logistic regression models assessed associations between knowledge, attitude, worry, and levels of anxiety and psychological distress.

**Results:** A total of 481 HCWs participated in this study. The majority of HCWs were female (73.6%) and aged 25–34 years (52.6%). More than half were nurses (55.7%) and had good knowledge of COVID-19 (86.3%). Over a third (37%) of HCWs reported moderate/severe psychological distress in the K10 measure and moderate/severe anxiety (32.3%) in the GAD-7, with frontline workers significantly reporting higher levels of anxiety (36%). Knowledge of COVID-19 did not predict anxiety and psychological distress, however, HCWs who believed COVID-19 was difficult to treat and those who perceived they were at high risk of infection had worse mental health outcomes. Worry about spreading COVID-19 to family, being isolated, contracting COVID-19 and feeling stigmatized had 1.8- to 2.5-fold increased odds of symptoms of mental health problems. Additionally, HCWs who felt the need for psychological support through their workplace showed increased odds of psychological distress.

**Conclusion:** HCWs in the UAE reported a high prevalence of psychological distress and anxiety while responding to the challenges of COVID-19. The findings from this study emphasize the public, emotional and mental health burden of COVID-19 and highlight the importance for health systems to implement, monitor, and update preventive policies to protect HCWs from contracting the virus while also providing psychological support in the workplace.

## Introduction

The Coronavirus disease 2019 (COVID-19) appeared in Wuhan, China, as a new respiratory illness caused by severe acute respiratory syndrome coronavirus 2 (SARS-CoV-2) [1]. On December 31, 2019, the Chinese CDC announced the emergence of a cluster of severe pneumonia cases of unrecognized etiology, which quickly crossed international borders [2]. The virus was declared a public health emergency by the World Health Organization (WHO) on January 30, 2020 and a global pandemic on March 11, 2020 [3]. Almost 15 months later, the COVID-19 virus has infected globally, an estimated 182 million cases, resulting in over 3 million deaths from 189 countries as of June 30, 2021 [4]. In the United Arab Emirates (UAE), 632,907 cases have been reported, and 1811 deaths were attributed to the illness during this period [5]. With ongoing community transmission, unpredictability, and uncertainty of disease control, the burden from this disease continues to rise, putting more individuals at risk of infection. Healthcare workers (HCW)s are at higher risk for COVID-19 infection due to their frontline work and exposure to COVID-19 infected patients [6–8]. Earlier reports identified Italy as the first European country outside of China to be severely hit by the pandemic with almost 10,000 HCWs being affected [9]. HCWs suffer a significant burden from COVID-19, with one study reporting nurses being the most commonly infected with an estimated prevalence of 11% [10]. In another study among infected symptomatic HCWs in Washington, United States, an overall prevalence of 5.3% has been reported [11].

Previous research has shown that historically, exposure to pandemics is accompanied by abnormal mental wellbeing in the general population and HCWs–ranging from mood disorders, anxiety symptoms, psychological distress to psychosis and cognitive impairments [12–15]. During the swine flu pandemic (2009–2010), a study among the United Kingdom population reported that 9.6 and 32.9% of the general public were either very or somewhat worried about potentially being infected by the disease, respectively [12]. Furthermore, in an ever-changing and unpredictable pandemic like COVID-19, HCWs are on the front lines of evaluating and managing affected persons throughout outbreaks. HCWs are more likely to acquire anxiety symptoms, psychological distress, and other mental health disorders while treating COVID-19 patients [12–15]. Furthermore, in the COVID-19 era, the immediate potential shift from healthcare provider to patient might lead to frustration, impotence, regulatory concerns, shame, and fear of discrimination among HCWs [9]. Heavy workload, fear of infection and spread to family, colleagues, and friends, concerns about quarantine and stigmatization, and heightened stress, anxiety, and depressive symptoms were among the negative psychological consequences experienced by HCWs during the 2003 SARS outbreak [12–15]. Recent studies have reported that HCWs in China, Saudi Arabia, Iran, Malaysia, Jordan, and South Asia have experienced abnormal mental wellbeing due to COVID-19 [16–24]. However, factors associated with mental issues such as psychological distress and anxiety among HCWs in the UAE are poorly understood. In China, psychological assistance services have been initiated to help the general population cope with stress, while the WHO has produced concise guidance to help frontline workers cope with stress [25]. However, evidence-based assessments and interventions to address the mental wellbeing of frontline workers in the COVID-19 era are crucial but lacking.

This study aims to quantify the magnitude of psychological distress and anxiety symptoms experienced by HCWs in the UAE as a result of COVID-19, as well as identify the relationship between socio-demographic factors, knowledge, beliefs, and concerns about COVID-19, and other factors associated with mental health issues experienced by HCWs.

## Methods

### Study Design

HCWs working in hospitals across the UAE between April 19 and May 3, 2020 were included in this cross-sectional study. We gathered information using the survey monkey platform to conduct an anonymous and self-reported online survey [26]. The study was approved by the University of Sharjah Research Ethics Committee (REC20-03-03-02) and the Ethics and Research Committee at the University Hospital- Sharjah (UHS-HERC-032-26032020). All participants provided informed consent electronically before completing the survey and were given the opportunity to opt out at any time.

### Participants and Sample Size

HCWs were randomly invited through the clinical services sections of three hospitals within the UAE. The hospitals were selected based on their collaborative relationship with the University of Sharjah and were from the emirates of Sharjah, Dubai and Abu Dhabi. An invitation and survey link were sent *via* email or WhatsApp message. The front page of the survey described the study objectives and assured confidentiality and anonymity. A study conducted in Saudi Arabia found that 26.5% of HCWs reported moderate depressive and anxiety disorders [24]. Given the similarities in HCW profiles between Saudi Arabia and the UAE, we used the same proportion for the calculation of sample size for this study. Using the proportion of moderate effect size of 26.5% and considering a two-sided test with an absolute sampling error of 0.05 and confidence level of 95%, a minimum sample size of 311 HCWs was calculated for this study. To account for non-response, attrition, and subgroup statistical analysis, the sample size was increased by 20%, making the minimum sample size required for this study 373 participants.

### Data Collection

A questionnaire was developed using modified questions from two previous studies investigating HCW concerns and worries with the A1/H1N1 outbreak [27] and, more recently, COVID-19 [21]. The questionnaire consisted of six parts. These included, 1) Demographic data: such as age, sex, profession, and workplace. HCWs were divided into four groups: Physicians (Consultant/specialist, general practitioner, and medical resident), nurses, allied staff (dentist, paramedics, pharmacists, laboratory personnel, and radiology technicians), and joint academic staff (full-time faculty, joint academic faculty). 2) Knowledge of and attitude to COVID-19: General questions about HCWs knowledge regarding the COVID-19 virus (prognosis, symptoms, transmission, treatment, prevention) and information sources. Knowledge was assessed using seven factual statements for which HCWs responded with “True,” “False,” or “I do not know.” A similar assessment of knowledge has been used in the literature [21, 27]. A score of >70% (5/7) defined a good level of knowledge of COVID-19, while a score below 70% was considered poor knowledge. Additionally, seven questions assessed HCWs attitude to COVID-19. Participants responded to each question using a 5-point Likert scale (consisting of strongly disagree, disagree, neither agree nor disagree, agree and strongly agree).

3) Anxiety: HCWs anxiety levels were assessed using the Generalized Anxiety Disorder 7-item (GAD-7) scale [28]. The scale consists of seven items with responses ranging from (0), not at all, to [3] nearly every day. Scores of 5, 10, and 15 were taken as the cut-off points for mild, moderate, and severe anxiety, respectively: 0–4 Minimal level of anxiety. 5–9 Mild level of anxiety, 10–14 Moderate level of anxiety, 15–21 Severe level of anxiety. A score of 10 or greater represents a reasonable cut point for identifying cases of anxiety with a sensitivity of 89% and specificity of 82%, internal consistency (Cronbach *α* = 0.92), and Test-retest reliability (intraclass correlation = 0.83) [28].

4) Psychological distress: This was assessed using the Kessler Psychological Distress (K10) scale [29]. The (K10) is a well-validated self-report clinical measure of psychological symptoms noted for its simplicity, ease of use, accessibility, and high predictability of psychological distress among adults [21]. The scale consists of 10 items, and the response to each question ranges from [1] none of the time to [5] all the time. The total score is the sum of scores obtained for these questions and are classified as; 10–19 Likely to be well, 20–24 Likely to have a mild disorder, 25–29 Likely to have a moderate disorder, 30–50 Likely to have a severe disorder [29, 30].

Other sections of the questionnaire covered; 5) Behavioral changes HCWs had made since the COVID-19 outbreak, such as restricting social contacts, whether their social contacts avoided them because they were considered high risk, or whether they had considered self-isolating from family because they believed they were high risk. Furthermore, HCWs reported 6) modifications in hygiene behaviors after the outbreak of the COVID-19 pandemic.

### Statistical Analysis

Data were coded and analyzed using the IBM Statistical Package for the Social Sciences (SPSS, version 24.0, Chicago, IL) software [31]. Demographic characteristics were described using frequency distributions and percentages. Using the Shapiro–Wilk’s test to measure normality of the data, the overall GAD-7 and K10 scores were not normally distributed and reported as medians with interquartile ranges. The Mann-Whitney *U* test and Kruskal-Wallis test explored associations between demographic characteristics and the GAD-7 and K10 scores between two or more groups. Whereas for categorical variables, differences in percentages were tested using Pearson Chi-square (*χ* ^2^). Multivariable logistic regression analysis was performed to identify potential risk factors for moderate/severe anxiety or moderate/severe psychological distress. Variables with a *p* < 0.2 in the univariate analysis and known risk factors of anxiety and psychological distress were included in the model using the enter method. The results were presented as odds ratios (ORs) with their 95% confidence intervals (CIs) after adjustment for the effects of potential confounders. HCWs who reported they were directly involved in the management of presumptive or confirmed COVID-19 cases were classified as frontline workers, while those without contact were considered second-line workers. A two-tailed *p* < 0.05 was considered statistically significant.

## Results

### Participants’ Characteristics

A total of 608 HCWs participated in the research. However, complete data for 481 HCWs was analysed, resulting in a 79% completeness rate for this study. The demographic and professional characteristics of HCWs are presented in Table 1. Nurses made up more than half of the participants, followed by physicians and other HCWs. Females accounted for 354 of the 481 HCWs. Young adults aged 25–34 years old made up more than half of HCWs (52.6%), followed by 35–44 years old (25.4%). Overall, 308 (64%) HCWs reported having interaction with a suspected or confirmed COVID-19 case and were classified as frontline workers.

**TABLE 1:** Summary demographic and professional characteristics of healthcare workers (*n* = 481). Psychological distress and anxiety levels among health care workers at the height of the COVID-19 pandemic in the United Arab Emirates, United Arab Emirates, 2020.

		N	%
**Profession**	Physician	108	22.2
	Nurse	268	55.7
	Allied health workers	73	15.2
	Auxiliary/Other workers	32	6.7
**Age (Years)**	18–24	43	8.9
	25–34	253	52.6
	35–44	122	25.4
	45–54	52	10.8
	55 and above	11	2.3
**Gender**	Female	354	73.6
	Male	127	26.4
**Area of work**	Inpatient wards	124	25.8
	Intensive Care Unit	24	5.0
	Isolation wards	24	5.0
	Emergency	96	20.0
	Hospital Pharmacy	20	4.2
	Hospital Laboratory	21	4.4
	Outpatient Clinics	79	16.4
	Dental Hospital/Clinic	20	4.2
	Radiology	23	4.8
	Other (University, Operating room, dialysis unit)	50	10.5
**In contact with any proven or suspected COVID-19 patients**			
No contact		173	36.0
Contact with both COVID-19 infected and suspected cases		112	23.3
Contact with COVID-19 patients		65	13.5
Contact with suspected COVID-19, but screening was negative		131	27.2
**Exposure classification**	Frontline workers	308	64.0
	Second-line workers	173	36.0
**Sources of COVID-19 information**	Hospital announcements	346	71.9
	Official statements or MOH press releases	330	68.6
	WHO website	367	76.3
	National media (TV, Radio)	264	54.9
	Social Media (Facebook, twitter, Instagram, WhatsApp)	291	60.5
	Other (CDC website and medical literature)	51	10.6
**HCWs concerns since emergence of COVID-19 pandemic**	I have restricted my social contacts (Yes)	442	91.9
	Family members/friends avoid contact with me (Yes)	282	58.6
	I am considering taking leave to avoid work (Yes)	185	38.5
	I have considered isolating myself from my family (Yes)	317	65.9

Nurses (69.2%) were the most common HCWs in contact with a suspected or confirmed COVID-19 case, followed by physicians (63.3%), allied health professionals (50.7%), and auxiliary staff (50.7%).). COVID-19 information was most usually sought from the WHO website (76.3%), followed by hospital announcements and official statements or news releases from the Ministry of Health and Prevention (MOHAP). In addition, several HCWs indicated they used social media sites (Facebook, Twitter, Instagram, and WhatsApp) to find COVID-19-related material. The majority of HCWs reported their social contacts were limited because they worked in a “high-risk” setting, and more than half said their relatives and friends avoided them because they worked in a “high-risk” environment (Table 1).

A higher proportion of allied health workers (68.5%) than physicians (63.0%) and nurses (56.3%) felt that their family members/friends were avoiding contact with them because they worked in a “high-risk” environment [χ^2^ (3, *n* = 481) = 8.62, *p* = 0.035]. Additionally, a higher proportion of frontline workers than second-line workers reported that they had restricted their social contacts (94.2 vs. 87.9%) [χ^2^ (1, *n* = 481) = 5.89, *p* = 0.015] or considered self-isolation (70.1 vs. 58.4%) [χ^2^ (1, *n* = 481) = 6.80, *p* = 0.009] because they considered themselves “high risk” (Data not presented in a table).

### HCW Knowledge, Attitude, and Perceived Sufficiency of Information Regarding COVID-19

As indicated in Table 2, almost all HCWs correctly answered knowledge questions about the COVID-19 virus’s symptoms, treatment, transmission, and risk; however, less than half of participants (46.8%) knew that COVID-19 patients can present with neurological symptoms. The average knowledge score (5.27 ± 0.75) was rated very high. We found that 86.3% of HCWs had overall good understanding of COVID-19 when we disaggregated knowledge scores using the specified cut-offs. The majority of participants believed their department had provided them with sufficient knowledge about COVID-19 symptoms, prognosis, treatment, contagion route, and preventative measures; nurses reported a much greater percentage of perceived sufficiency of information. Almost all HCWs reported they had changed their behaviors since the outbreak of the COVID-19 pandemic. Almost all HCWs had increased their use of universal measures such as face masks, hand sanitizer, and handwashing. In addition, most HCWs reported fewer social interactions, handshakes, and visits to crowded venues such as malls (Supplementary Figure S1).

**TABLE 2:** Healthcare workers’ knowledge and perceived sufficiency of information about COVID-19. Psychological distress and anxiety levels among health care workers at the height of the COVID-19 pandemic in the United Arab Emirates, United Arab Emirates, 2020.

						N	%
**Knowledge of COVID-19 virus^#^**							
It can be transmitted by droplets from patient’s coughing/sneezing						474	98.5
It can be transmitted by contact with patient tools and then face						470	97.7
It can cause severe respiratory symptoms						471	97.9
It can cause neurological symptoms						225	46.8
Healthcare workers are at higher risk						477	99.2
There is no vaccine						448	93.1
There is no specific treatment						419	87.1
**Knowledge Score 0–7 (Mean ± SD)** 5.27 ± 0.75							
**Knowledge Category**	Poor					66	13.7
	Good					415	86.3
			**Physician**	**Nurse**	**Allied**	**Auxiliary**	**^a^ *p* Value**
I believe I have received sufficient information from my department about COVID-19*	Symptoms		96 (88.9)	251 (93.7)	68 (93.2)	26 (81.3)	0.137
	Prognosis		77 (71.3)	233 (86.9)	60 (82.2)	20 (62.5)	**0.001**
	Treatment		74 (68.5)	213 (79.5)	48 (65.8)	21 (65.6)	**0.005**
	Transmission		95 (88.0)	255 (95.1)	67 (91.8)	26 (81.3)	**0.015**
	Prevention		84 (77.8)	252 (94.0)	68 (93.2)	23 (71.9)	**≤0.001**
Attitude to and beliefs of COVID-19*							
		**N (%)**	**Physician**	**Nurse**	**Allied**	**Auxiliary**	**^a^ *p* Value**
I believe my risk for infection with COVID-19 is high		406 (84.4)	95 (88.0)	234 (87.3)	58 (79.5)	19 (59.4)	**0.001**
I believe being infected with COVID-19 will have major consequences on my health		377 (78.4)	72 (66.7)	230 (85.5)	54 (74.0)	21 (65.6)	**0.001**
I believe that infection with COVID-19 is difficult to be treated		315 (65.5)	58 (53.7)	201 (75.0)	40 (54.8)	16 (50.0)	**≤0.001**
I feel that my workplace is well prepared for the COVID-19 pandemic		328 (68.2)	60 (55.6)	195 (72.8)	56 (76.7)	17 (53.1)	**0.001**
I think it is important to offer psychological support regarding concerns about COVID-19 in the workplace		388 (80.7)	82 (75.9)	222 (82.8)	58 (79.5)	26 (81.3)	0.303
I believe public fear about COVID-19 is justifiable/appropriate		436 (90.6)	91 (84.3)	248 (92.5)	67 (91.8)	30 (93.8)	**0.019**
I believe the fear about COVID-19 is dysfunctional (e.g. it has caused unnecessary absence from schools/universities etc.)		212 (44.1)	41 (38.0)	134 (50.0)	28 (88.4)	9 (28.1)	**≤0.001**

^#^indicates those who answered the statement correctly.

*indicates those who strongly agreed or agreed with statement.

^a^Using Chi-square analysis and significant at *p* < 0.05.

The attitudes and views of HCWs concerning COVID-19 are shown in Table 2. The majority of HCWs believed their chances of contracting the SARS-CoV-2 virus were high, with physicians having a higher perception of risk than other HCWs [χ^2^ (6, *n* = 481) = 23.98, *p* = 0.001]. Nurses were far more likely than other HCWs to feel that becoming infected would have serious health effects (85.5%), [χ^2^ (6, *n* = 481) = 22.58, *p* = 0.001], and that it would be difficult to treat (75.0%), [χ^2^ (6, *n* = 481) = 29.28, *p* = 0.001]. A much lower percentage of auxiliary employees (53.1%) reported their workplace was prepared for the COVID-19 pandemic [χ^2^ (6, *n* = 481) = 22.59, *p* = 0.001]. Almost all HCWs agreed that psychological support at the workplace was important, with no difference amongst them. Nearly all participants believed that the public’s concern of COVID-19 was justified, while only 44% thought it was dysfunctional.

### Scores of Worry, Measurements of Mental Health Outcomes, and Associated Factors

Table 3 reports the K10 psychological distress score and GAD-7 anxiety scores, as well as the median (IQR) scores and mean ranks for levels of worry. Worry was assessed on a scale of one to five, with five indicating the greatest level of concern. Overall, HCWs’ median worry scores for developing COVID-19, transmitting it to their families, and being isolated if they contracted COVID-19 were 4 (extremely worried). The Kruskal Wallis H test assessed differences in HCWs’ worry levels. The median anxiety scores for contracting COVID-19 [χ^2^ (3) = 5.905, *p* = 0.116] and transferring it to family [χ^2^ (3) = 2.702, *p* = 0.558] did not differ statistically. However, when it came to fear of COVID-19-related isolation, there was a statistically significant difference between HCW groups [χ^2^ (3) = 7.943, *p* = 0.047]. Post hoc analysis showed higher levels of anxiety in nurses, with statistically significant differences in mean rank worry scores between nurses and physicians (*p* = 0.024) and nurses and auxiliary staff (*p* = 0.043). Females were more concerned than males about being isolated due to COVID-19 (*U* = 18703, z = −2.941, *p* = 0.003). Females and frontline HCWs were also more concerned than men and second-line HCWs about passing COVID-19 to their families (*U* = 19839, z = −2.105, *p* = 0.035) and (*U* = 23332, z = −2.432, *p* = 0.015), respectively.

**TABLE 3:** Worry, psychological distress and anxiety among healthcare workers by profession, gender and exposure. Psychological distress and anxiety levels among health care workers at the height of the COVID-19 pandemic in the United Arab Emirates, United Arab Emirates, 2020.

			Profession					Gender			Work exposure		
Median (IQR)			Mean rank					Mean rank			Mean rank		
Worry 1= not worried - 5 = extremely worried			Physician	Nurse	Allied	Auxiliary	*p Value	Female	Male	^b^ *p* Value	Frontline	Second line	^b^ *p* Value
Worried may contract COVID-19^a^		4 (3,5)	219.13	253.27	227.95	241.80	0.116	247.08	224.04	**0.094**	247.08	230.17	0.181
Worried may transmit COVID-19 to family^a^		4 (4,5)	226.30	245.35	249.47	234.88	0.558	248.46	220.21	**0.035**	251.78	221.81	**0.015**
Worried about isolation due to COVID-19^a^		4 (3,5)	220.77	254.87	235.98	204.56	**0.047**	251.67	211.27	**0.003**	245.59	232.82	0.312
K10, Psychological distress Score		19 (13,30)	241.16	241.46	249.15	217.97	0.769	248.93	218.89	**0.036**	251.18	222.87	**0.032**
GAD-7, Anxiety Score		8 (3,13)	245.20	242.27	224.29	254.28	0.688	255.82	199.69	**<0.001**	248.63	227.42	0.107
			**Profession n (%)**					**Gender n (%)**			**Work exposure n (%)**		
		**N (%)**	**Physician**	**Nurse**	**Allied**	**Auxiliary**	^*#*^ ***p-value***	**Female**	**Male**	^*#*^ ***p-value***	**Frontline**	**Second line**	^*#*^ *p-value*
K10 Scale	Normal	245 (51.1)	53 (49.1)	143 (53.4)	33 (45.2)	17 (53.1)	0.812	174 (49.2)	72 (56.7)	0.15	146 (47.4)	100 (57.8)	0.112
	Mild	57 (11.9)	14 (13.0)	29 (10.8)	12 (16.4)	2 (6.3)		42 (11.9)	15 (11.8)		38 (12.3)	19 (11.0)	
	Moderate	56 (11.6)	12 (11.1)	29 (10.8)	9 (12.3)	6 (18.8)		48 (13.6)	8 (6.3)		36 (11.7)	20 (11.6)	
	Severe	122 (25.4)	29 (26.9)	67 (25.0)	19 (26.0)	7 (21.9)		90 (25.4)	32 (25.2)		88 (28.6)	34 (19.7)	
GAD-7 Scale	Minimal	166 (34.5)	36 (33.3)	93 (34.7)	26 (35.6)	11 (34.4)	0.826	102 (28.8)	64 (50.4)	**<0.001**	100 (32.5)	66 (38.2)	**0.015**
	Mild	160 (33.3)	36 (33.3)	89 (33.2)	28 (38.4)	7 (21.9)		126 (35.6)	34 (26.8)		97 (31.5)	63 (36.4)	
	Moderate	63 (13.1)	16 (14.8)	32 (11.9)	9 (12.3)	6 (18.8)		50 (14.1)	13 (10.2)		42 (13.6)	21 (12.1)	
	Severe	92 (19.2)	20 (18.5)	54 (20.1)	10 (13.7)	8 (25.0)		76 (21.5)	16 (12.6)		69 (22.4)	23 (13.3)	

*Using Kruskal-Wallis test and significant at *p* < 0.05.

^#^Using Chi-Square test and significant at *p* < 0.05.

^a^Worry measured on Likert Scale where 1 = Not worried - 5 = Very worried.

^b^Using Mann Whitney U test and significant at *p* < 0.05.

**FIGURE 1:**
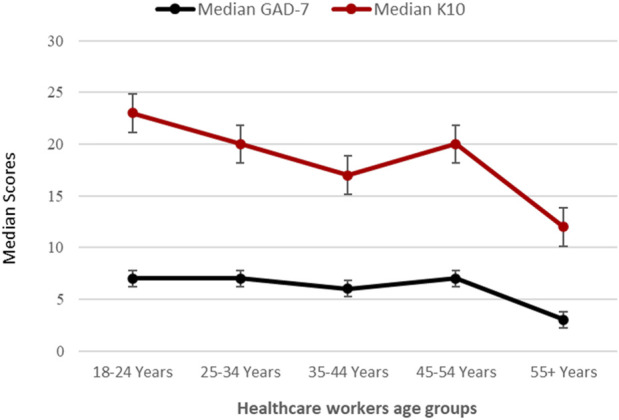
Healthcare workers’ age groups and their corresponding median raw anxiety and psychologic distress scores. Psychological distress and anxiety levels among health care workers at the height of the COVID-19 pandemic in the United Arab Emirates, United Arab Emirates, 2020.

Table 3 shows that the median K10 psychological distress and GAD-7 anxiety scores for all HCWs were 19.0 (13.0–30.0) and 8.0 (3.0–13.0), respectively. The mean rank scores for psychological distress and anxiety were not significantly different across the various HCW occupations. Females [20.0 vs. 16.0; (*U* = 19671, z = −2.092, *p* = 0.036)] and frontline HCWs [20.0 vs. 17.0; [*U* = 23506, z = −2.146, *p* = 0.032)] had substantially higher median (IQR) scores on the K10 for psychological distress. Females scored higher on the GAD-7 anxiety scale (7.0 vs. 4.0) (U = 17223, z = −3.196, *p* < 0.001). A substantial proportion of HCWs reported symptoms of psychological distress (48.9%) and generalized anxiety disorder (65.5%). More than one-third of HCWs (37.0%) reported moderate to severe psychological distress, and one-third of HCWs (32.3%) reported moderate to severe anxiety. No differences were found between levels of psychological distress or anxiety among the different HCW professions, gender, or being frontline or second-line workers for psychological distress. As demonstrated in Table 3, frontline workers reported significantly greater levels of moderate and severe anxiety than second-line workers and substantially more females than males experienced moderate and severe anxiety (*p* ≤ 0.001). Participants’ median scores on the K10 and GAD-7 scales declined dramatically as they became older. Younger HCWs showed substantially higher K10 [χ^2^ (4) = 15.954, *p* = 0.003] and GAD-7 scores than older age groups [χ^2^ (4) = 14.269, *p* = 0.006] (Figure 1).

### Risk Factors for Severe Psychological Distress and Anxiety

Age, being a frontline worker, attitude towards COVID-19, increasing levels of worry associated with COVID-19, restricting social contacts, friends and family avoiding contact, considering a leave of absence from work, and thoughts of isolating were all significantly associated with moderate and severe psychological distress in the univariate analysis. However, when controlling for age, gender, profession, work position, knowledge, attitude, worry, and behavioral changes; being concerned about contracting the virus (aOR 1.61; 95% CI 1.10–2.36); spreading it to family members (aOR 1.79; 95% CI 1.24–2.58); believing it was difficult to treat (aOR 1.67; 95% CI 1.18–2.35); believing it was important for psychological support at the workplace (aOR 1.64; 95% CI 1.15–2.35) and concerns about being isolated due to COVID-19 infection (aOR 1.37; 95% CI 1.02–1.83) significantly predicted higher levels of psychological distress in HCWs (Table 4). Similarly, gender, being a frontline worker, attitudes towards COVID-19, higher levels of worry, family and friends avoiding contact, considering a leave of absence, and isolation were associated with moderate and severe generalized anxiety in the univariate analysis (Table 5). However, with further analysis controlling for these factors; being female (aOR 1.93; 95% CI 1.15–4.11), perceived high risk of infection (aOR 1.68; 95% CI 1.05–2.70), perceived readiness of their workplace for the pandemic (aOR 0.63; 95% CI 0.49–0.82), being worried about contracting SARS-CoV-2 (aOR 2.65; 95% CI 1.64–4.30) and feeling that family members and friends were avoiding them (aOR 1.65; 95% CI 1.01–2.68), all significantly predicted higher levels of generalized anxiety disorder among HCWs.

**TABLE 4:** Risk factors for moderate/severe psychological distress (K10 scores) identified by multivariable logistic regression analysis. Psychological distress and anxiety levels among health care workers at the height of the COVID-19 pandemic in the United Arab Emirates, United Arab Emirates, 2020.

Variables	No. of cases/No. Total cases (%)	Univariate OR (95% CI)	*p* Value	Adjusted OR (95% CI)^a^	*p* Value^a^
**Age (Years)**					
18–44	164/418 (39.2)	2.26 (1.21–4.22)	**0.001**	1.01 (0.47–2.37)	0.89
45 and above	14/63 (22.2)	1		1	
**Gender**					
Female	138/354 (39.0)	1.39 (0.90–2.14)	0.139	1.13 (0.60–2.13)	0.70
Male	40/127 (31.5)	1		1	
**Profession**					
Physician	41/108 (38.0)	1		1	
Nurse	96/268 (35.8)	0.91 (0.58–1.45)	0.70	0.75 (0.38–1.50)	0.42
Allied health workers	28/73 (38.4)	1.02 (0.55–1.87)	0.96	0.95 (0.42–2.14)	0.91
Auxiliary/Other workers	13/32 (40.6)	1.12 (0.50–2.50)	0.79	1.08 (0.53–2.21)	0.83
**Work exposure**					
Frontline workers	124/308 (40.3)	1.49 (1.01–2.20)	**0.05**	1.68 (0.96–2.95)	0.07
Second-line workers	54/173 (31.2)	1		1	
**Knowledge of COVID-19**					
Poor	26/66 (39.4)	1.13 (0.66–1.92)	0.66	1.01 (0.53–2.21)	0.83
Good	152/415 (36.6)	1		1	
**Attitude towards COVID-19^b^**					
My risk for infection is high		1.47 (1.13–1.91)	**0.004**	0.85 (0.58–1.23)	0.38
COVID-19 will have major consequences on my health		1.43 (1.12–1.83)	**0.005**	0.70 (0.47–1.05)	0.09
COVID-19 difficult to treat		1.71 (1.36–2.15)	**<0.001**	1.67 (1.18–2.35)	**0.004**
Work prepared for pandemic		0.92 (0.76–1.10)	0.36	0.73 (0.57–0.94)	**0.013**
Important to offer psychological support at work		1.95 (1.46–2.60)	**<0.001**	1.64 (1.15–2.35)	**0.007**
Public fear is justifiable		1.39 (1.02–1.88)	**0.035**	2.24 (0.86–5.84)	0.10
**Worry associated with COVID-19^c^**					
Worried may contract virus		2.20 (1.76–2.75)	**<0.001**	1.61 (1.10–2.36)	**0.014**
Worried may spread COVID-19 to family members		3.00 (2.25–4.00)	**<0.001**	1.79 (1.24–2.58)	**0.037**
Worried about being isolated due to COVID-19 infection		2.22 (1.79–2.77)	**<0.001**	1.37 (1.02–1.83)	**0.048**
**I have restricted my social contacts because my work environment is considered “high-risk”**					
Yes	171/442 (38.7)	2.89 (1.25–6.68)	**0.013**	1.81 (0.49–6.71)	0.37
No	7/39 (17.9)	1		1	
**I feel that my family members/friends avoid contact with me, because I work in a “high-risk” environment**					
Yes	117/282 (41.5)	1.60 (1.09–2.35)	**0.016**	2.68 (1.53–4.71)	**0.001**
No	61/199 (30.7)	1		1	
**Lately I am so concerned about COVID-19, that I am considering taking leave to avoid going to work**					
Yes	92/185 (49.7)	2.42 (1.65–3.54)	**<0.001**	2.68 (1.55–4.65)	**<0.001**
No	86/296 (29.1)	1		1	
**Lately, I have thought about isolating myself from my family because I consider myself “high risk”**					
Yes	142/317 (44.8)	2.89 (1.88–4.44)	**<0.001**	1.61 (0.86–3.00)	0.13
No	36/164 (22.0)	1		1	

^a^Adjusted for: age, gender, profession, work position, level of knowledge, attitude, worries regarding COVID-19 and behavioral changes since COVID-19.

^b^Attitude: ranges from 1 to 5 (1 = Strongly Disagree to 5 = Strongly Agree).

^c^Level of worry: ranges from 1 to 5 (1 = not at all, 5 = extremely worried) Model Fit: Hosmer and Lemeshow test (X^2^ 18.42, df = 8; *p* = 0.31); −2Log likelihood 430.62.

**TABLE 5:** Risk factors for moderate/severe generalized anxiety disorder (GAD-7 scores) Identified by multivariable logistic regression analysis. Psychological distress and anxiety levels among health care workers at the height of the COVID-19 pandemic in the United Arab Emirates, United Arab Emirates, 2020.

Variables	No. of cases/No. Total cases (%)	Univariate OR (95% CI)	*p* Value	Adjusted OR (95% CI)^a^	*p* Value
**Age (Years)**					
18–44	134/418 (32.1)	1.06 (0.60–1.86)	0.84	1.37 (0.68–2.75)	0.38
45 and above	21/63 (33.3)	1		1	
**Gender**					
Female	126/354 (35.6)	1.87 (1.17–2.98)	**0.009**	1.93 (1.15–4.11)	**0.029**
Male	29/127 (22.8)	1		1	
**Profession**					
Physician	36/108 (33.3)	1		1	
Nurse	86/268 (32.1)	0.95 (0.59–1.52)	0.82	1.26 (0.56–2.84)	0.58
Allied health workers	19/73 (26.0)	0.70 (0.36–1.36)	0.30	1.36 (0.49–3.78)	0.56
Auxiliary/Other workers	14/32 (43.8)	1.56 (0.70–3.48)	0.28	2.51 (0.77–8.26)	0.13
**Work exposure**					
Frontline workers	111/308 (36.0)	1.65 (1.09–2.50)	**0.017**	1.35 (0.72–2.53)	0.34
Second-line workers	44/173 (25.4)	1		1	
**Knowledge of COVID-19**					
Poor	16/66 (24.2)	1.57 (0.87–2.86)	0.14	1.84 (0.94–3.60)	0.08
Good	139/415 (33.5)	1		1	
**Attitude towards COVID-19^b^**					
My risk for infection is high		2.07 (1.46–2.93)	**<0.001**	1.68 (1.05–2.70)	**0.030**
COVID-19 will have major consequences on my health		1.32 (1.01–1.73)	**0.04**	0.48 (0.29–0.73)	**0.001**
COVID-19 difficult to treat		1.64 (1.28–2.11)	**<0.001**	1.56 (1.10–2.32)	**0.028**
Work prepared for pandemic		0.74 (0.61–0.90)	**0.003**	0.63 (0.49–0.82)	**<0.001**
Important to offer psychological support at work		1.77 (1.30–2.43)	**<0.001**	1.34 (0.93–1.93)	0.12
**Worry associated with COVID-19^c^**					
Worried may contract virus		2.98 (2.30–3.87)	**<0.001**	2.65 (1.64–4.30)	**<0.001**
Worried may spread COVID-19 to family members		2.45 (1.86–3.24)	**<0.001**	0.91 (0.57–1.46)	0.69
Worried about being isolated due to COVID-19 infection		2.14 (1.71–2.69)	**<0.001**	1.21 (0.80–1.84)	0.37
**I have restricted my social contacts because my work environment is considered “high-risk”**					
Yes	146/442 (33.0)	1.64 (0.76–3.55)	0.21	2.96 (0.63–13.87)	0.17
No	9/39 (23.1)	1		1	
**I feel that my family members/friends avoid contact with me, because I work in a “high-risk” environment**					
Yes	103/282 (36.5)	1.63 (1.09–2.42)	**0.017**	1.65 (1.01–2.68)	**0.045**
No	52/199 (26.1)	1		1	
**Lately I am so concerned about COVID-19, that I am considering taking leave to avoid going to work**					
Yes	75/185 (40.5)	1.84 (2.25–2.72)	**0.002**	1.78 (0.96–3.30)	0.07
No	80/296 (27.0)	1		1	
**Lately, I have thought about isolating myself from my family because I consider myself “high risk”**					
Yes	119/317 (37.5)	2.14 (1.38–3.30)	**0.001**	1.68 (0.72–2.25)	0.40
No	36/164 (22.0)	1		1	

^a^Adjusted for: age, gender, profession, work position, level of knowledge, attitudes, worries regarding COVID-19 and behavioral changes since COVID-19.

^b^Attitude: ranges from 1 to 5 (1 = Strongly Disagree to 5 = Strongly Agree).

^c^Level of worry: ranges from 1 to 5 (1 = not at all, 5 = extremely worried) Model Fit: Hosmer and Lemeshow test (X^2^ 11.39, df = 8; *p* = 0.180); −2Log likelihood 356.33.

## Discussion

This study shows that COVID-19 has a considerable impact on the mental health of HCWs in the UAE. Anxiety disorders were reported by over two-thirds of HCWs, and nearly half experienced signs of psychological distress, with a large proportion experiencing severe distress and anxiety. Females in our study reported higher levels of worry associated with COVID-19. Furthermore, a higher percentage of female HCWs and frontline workers experienced moderate/severe anxiety, which decreased as they got older. Concerns about COVID-19 spreading to family members and the prospect of seclusion were determined to be independent risk factors for moderate/severe psychological distress. Female gender, stigmatization and avoidance by family/friends, and the fear of contracting COVID-19 were all found to be independent risk factors for moderate/severe generalized anxiety disorder.

In our study, a significant number of HCWs showed signs of psychological discomfort. These findings are congruent with research from Saudi Arabia, China, Italy, and the United States, which found that 31.3–71.5% of HCWs in these countries are experiencing psychological distress [22, 32–34]. Similarly, the high prevalence of generalized anxiety disorder among HCWs in our study is consistent with reports from Saudi Arabia [21, 24], China [22], Poland [35], and Italy [36]. HCWs are at the forefront of care. Fear and uncertainty about becoming infected, the possibility of spreading the virus to their family, the possibility of death, and the lack of a recognized cure for the disease are all likely to contribute to high distress and anxiety [37]. Providing medical care in the midst of a global pandemic is frightening and stressful. Concerns about their inability to regulate the work environment in which COVID-19 patients are treated, changes in how clinical treatment is delivered, and feelings of isolation may also be sources of psychological distress and anxiety [22, 38].

We found that being a woman was linked to higher levels of anxiety and psychological suffering. Similar findings were found in recent research examining factors related to mental health outcomes among HCWs exposed to COVID-19 [22, 39–41]. Women are more likely than men to experience psychological disorders as a result of a complex combination of biological, social, and gender role requirements [41, 42]. Gender-informed policies, on the other hand, are critical during a pandemic to address and modify support services specifically for women. The state of one’s mental health is also related to one’s age. According to previous studies, younger age groups are more likely to experience worry and stress [24, 40, 43]. The current study indicated that median anxiety and distress levels varied with age, with lower scores among the older age group, which is consistent with previous research. An earlier investigation of HCWs found no correlation between age and emotional stress caused by COVID-19 [44]. This could be because the study was carried very early in the COVID-19 pandemic, when little was known about the disease, regardless of the HCWs’ age.

COVID-19 was well-understood by the HCWs in our study. Few studies have looked into the role of knowledge and other factors among HCWs, as well as their relationship to distress. Knowledge of COVID-19 and COVID-19 protective measures were found to be major determinants in medical staff resilience in a previous study [22]. In contrast, a lack of clarity might lead to increased levels of psychological suffering [45]. During the A/H1N1 influenza pandemic, a study on HCW worries and perceived psychological distress found that perceived sufficiency of prognosis information was linked to lower levels of worry [27] while another study found that well-informed HCWs in the UAE with evidence-based understanding were more likely to remain vigilant at all times, reducing the risks to themselves and their patients [46]. HCWs in our study had excellent levels of knowledge of COVID-19, which did not influence their mental health negatively. However, concerns about spreading COVID-19 to family members and feelings of isolation, were risk factors for moderate/severe psychological distress, confirming previous findings that HCWs involved in the direct care of COVID-19 patients had a higher risk of depression, anxiety, and distress [22]. These findings suggest that HCWs, particularly those with a higher perception of infection risk, require more psychological assistance, including psychological therapy that addresses specific worries about disease and spreading. Ultimately, how HCWs are seen by others, such as facing stigmatisation from their families and friends, can cause psychological discomfort in them. A recent study reported that stigma encountered by HCWs during the COVID-19 response was significantly associated with their risk of experiencing symptoms of anxiety [29]. Similarly, in a large-scale study of 10,511 HCWs, 49% experienced social stigmatization and 31% ostracism by family members during the SARS outbreak in Singapore [47].

Although HCWs in our study believed their workplace was adequately prepared for the pandemic, they also stressed the significance of receiving psychological support from their employers addressing their concerns about COVID-19. This belief also predicted moderate to severe psychological distress among HCWs. This emphasizes the importance of health systems implementing, monitoring, and updating preventive policies to protect HCWs from contracting the virus while also providing psychological support in the workplace. According to a recent study, an online technology service was used to provide an intervention for HCWs. A multidisciplinary team of doctors, psychologists, psychiatrists, and social workers were incorporated into online programs to give psychological mediation to patients, their family members, and HCWs in this novel model from a Chinese hospital. The basic idea behind this endeavor was to combine and expand on current support networks by combining psychiatric therapies and subsequent rehabilitation with internet technologies [48]. During the COVID-19 pandemic, a similar approach in the UAE would be extremely beneficial and will enhance existing support systems; in addition to health authorities and government bodies continuously publishing guidelines and notifications for emergency psychological crisis interventions and providing psychological assistance hotlines.

This study has several limitations. First, it is limited in scope. The participants were from only three hospitals across the UAE, limiting the generalizability of our findings. Second, the study was carried out during the early period of the outbreak and lacked longitudinal follow-up. Because of the increasingly tiring pandemic, continuous COVID-19 cases, new mutations, and virus variants, the mental health symptoms of HCWs could vary and become more severe with the recent surge in COVID-19 cases in some regions. Additionally, this study was unable to distinguish pre-existing mental issues versus new ones regarding COVID-19 nor determine whether nationality predicted mental health outcomes. Lastly, there could be information and recall bias due to the online self-report questionnaire. However, despite these limitations, our study provides information about the immediate psychological responses of HCWs in the UAE to the COVID-19 pandemic. It identified several factors that could affect the mental health and practices of HCWs in the UAE and provides baseline data to inform future longitudinal studies and policymaker decisions.

### Conclusion

In response to the problems of COVID-19, HCWs in the UAE reported a significant prevalence of psychological distress and anxiety. The findings from this study will assist and act as a reference for various organizations to quickly implement different psychological interventions for HCWs. The emotional and physical burden of COVID-19 may have detrimental and perhaps lasting effects on mental health and wellbeing in a place like the UAE, where most HCWs are expatriates. Government officials should establish multidisciplinary mental health units both locally and nationally to examine psychological health issues (stress, depression, and anxiety) and provide psychological assistance to those who need it.

## Data Availability

The datasets used and/or analyzed during the current study are available from the corresponding authors upon reasonable request.
