# Supplementary figures and images for: Psychological Distress and Anxiety Levels Among Health Care Workers at the Height of the COVID-19 Pandemic in the United Arab Emirates
Source: Int J Public Health. 2021 Nov 11;66:1604369. doi: 10.3389/ijph.2021.1604369 (PMC8615074; doi:10.3389/ijph.2021.1604369)

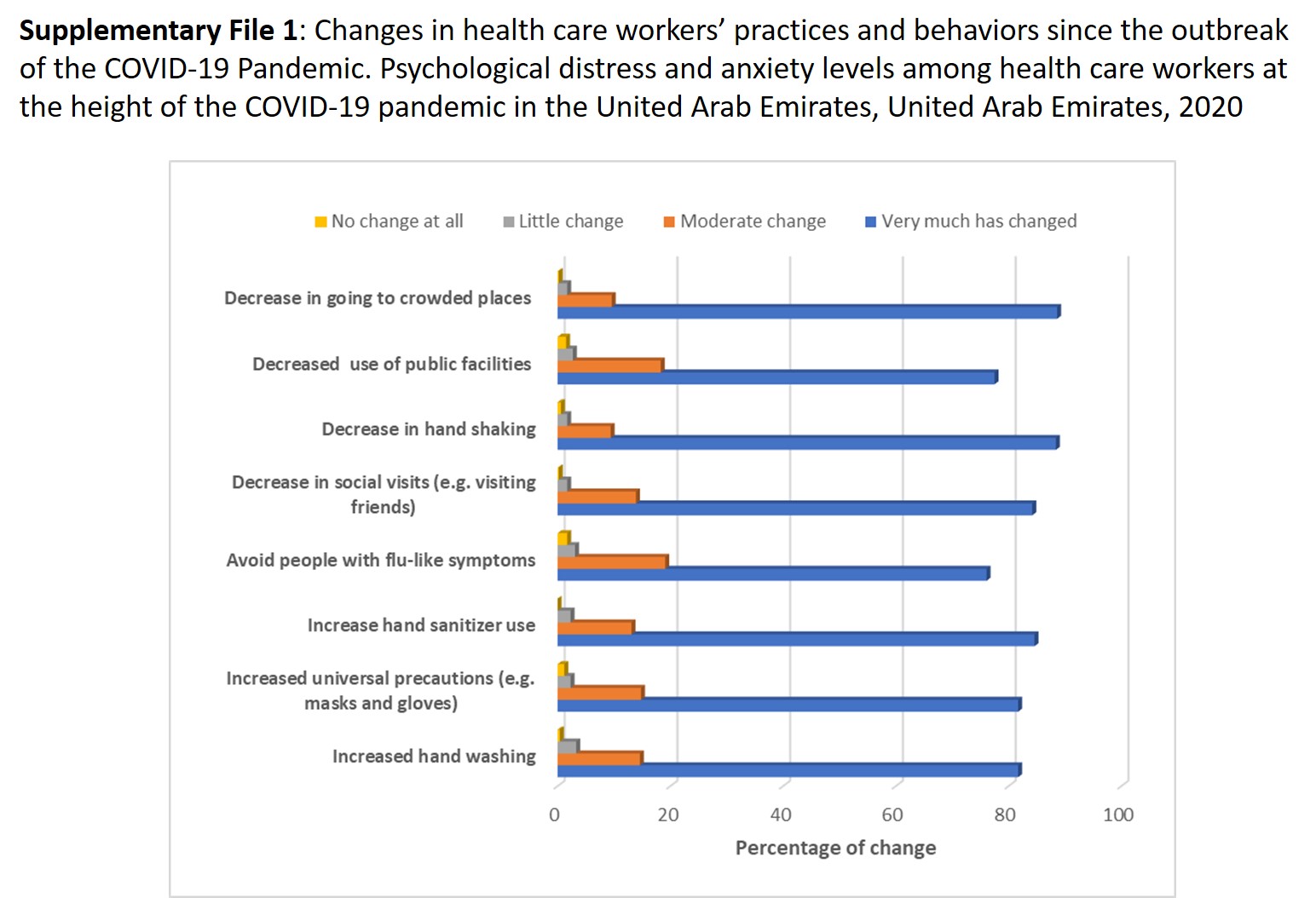

Supplement: Supplementary file 1 [file Image1.jpg]
